# Differential impacts of physical activity volume and intensity on blood lead levels in children and adolescents: a cross-sectional study

**DOI:** 10.1186/s12889-025-23333-8

**Published:** 2025-06-02

**Authors:** Shengrong Ouyang, Yan Yin, Yuanyuan Li, Jianxin Wu, Zhuo Liu

**Affiliations:** 1 Department of Biochemistry and Immunology, Capital Institute of Pediatrics, No. 2 Yabao Road, Chaoyang District, Beijing, 100020 China; 2 Department of Integrated Early Childhood Development, Capital Institute of Pediatrics, Beijing, China; 3 Beijing Municipal Key Laboratory of Child Development and Nutriomics, Beijing, China; 4 Beijing TongRen Hospital, Capital Medical University, Beijing, China

**Keywords:** Physical activity, Lead exposure, Blood lead, Accelerometer, NHANES

## Abstract

**Background:**

Previous studies on physical activity (PA) and blood lead levels (BLLs) have relied on self-reported data, conflating PA volume and intensity. This study aimed to disentangle their independent effects and test the hypothesis that higher PA volume increases BLLs via environmental lead exposure, while higher PA intensity reduces BLLs through enhanced excretion.

**Methods:**

We used data from the 2011–2014 National Health and Nutrition Examination Survey (NHANES) to quantify PA in 3,249 U.S. children and adolescents (aged 3–19 years) using two metrics based on monitor-independent movement summary units (MIMS): average daily MIMS for volume and peak 60-minute MIMS for intensity. BLLs were natural log-transformed (ln-BLL) for analysis. Weighted multivariable linear regression, general additive models, mediation analysis, and Bayesian kernel machine regression (BKMR) were employed to explore associations.

**Results:**

PA volume showed a significant positive association with ln-BLL (β = 0.025, *p* < 0.001), with a threshold effect at 19,700 MIMS. In contrast, PA intensity exhibited a non-significant negative correlation with BLL (β = -0.027, *p* = 0.156). Mediation analysis indicated that outdoor time explained 32.8% of the PA volume–BLL association. BKMR revealed cumulative effects of co-exposures on BLL trends. Sex-stratified analyses showed that males had higher BLLs at similar PA volume levels, while females experienced greater reductions in BLLs with increased PA intensity.

**Conclusion:**

Our findings support a dual-pathway model: greater PA volume may elevate BLL—particularly in males—while higher intensity may reduce BLL—especially in females. These results underscore the need for PA guidelines that balance volume and intensity, optimize sweat-promoting exercise in low-lead environments, and inform interventions to safeguard the health of children and adolescents against lead exposure.

**Supplementary Information:**

The online version contains supplementary material available at 10.1186/s12889-025-23333-8.

## Background

Lead is a pervasive environmental toxicant that poses significant health risks, particularly to children and adolescents [1]. Elevated blood lead levels (BLLs) during childhood have been associated with a spectrum of adverse health outcomes, including cognitive deficits, behavioral problems, and impaired growth [2–6]. The vulnerability of the developing nervous system to lead exposure underscores the importance of understanding and mitigating the risks associated with BLLs in young populations.

The historic use of leaded gasoline additives, paints, and industrial processes has generated persistent reservoirs of lead in soil, household dust, water, and ambient air [1]. Inorganic lead is remarkably stable, with soil-bound lead remaining bioavailable for decades after deposition [7]. Despite global efforts to phase out lead-based products, residual contamination still disproportionately affects communities near industrial zones and aging infrastructure.

Physical activity (PA) is widely acknowledged for its beneficial effects on overall health and well-being. It is particularly crucial to children and adolescents, who stand to gain substantial benefits from regular physical exercise, such as improved cardiovascular fitness, enhanced mental health, and reduced risk of chronic diseases [8, 9]. However, PA may paradoxically modulate lead exposure through multiple pathways. First, increased respiratory rates during exercise amplify the inhalation of airborne lead particles, especially in polluted environments [10]. Second, PA-induced movement can re-suspend settled soil and dust particles: field studies demonstrate that even light walking on lead-contaminated surfaces sharply elevates inhalable lead concentrations in the breathing zone [7]. Conversely, vigorous PA may enhance lead elimination through sweating. A systematic review suggests that sweat lead concentrations in individuals with elevated body burdens often exceed those in plasma or urine; in rare cases, daily dermal excretion via sweat during intense exercise (e.g., endurance rowing) may rival urinary lead elimination [11].

The relationship between PA and BLL remains complex and incompletely resolved. Some studies have suggested that certain types of PA, particularly those involving outdoor environments with potential lead contamination, may increase the risk of lead exposure and subsequent elevations in BLL [12, 13]. On the other hand, other research has suggested that PA may potentially lower BLLs, possibly by enhancing the excretion of lead through sweat [11, 14, 15]. This inconsistency emphasizes the importance of further investigating the relationship between PA and lead exposure.

Most evidence on the relationship between PA and BLL comes from studies using self-reported levels of PA, such as the one conducted by Rhie et al. [13]. This method is susceptible to recall and response biases, which may overestimate or underestimate actual PA energy expenditure and rates of inactivity. While self-report instruments are valuable tools for surveillance and epidemiological research, they are limited compared to device-based assessments, which offer more precise depictions of movement patterns and stronger associations with health outcomes [16]. Device-based measurements (e.g., accelerometers) address these limitations by providing objective, minute-by-minute PA data with superior precision and stronger associations with health outcomes [17, 18]. Yet, no studies to date have explicitly disentangled the distinct effects of PA volume (i.e., cumulative daily activity) and intensity (i.e., activity magnitude) on BLLs.

We hypothesize that high PA volume increases BLLs via increased inhalation and ingestion of resuspended lead dust, while high PA intensity may modestly enhance sweat-mediated lead excretion. To test this, we analyzed data from the National Health and Nutrition Examination Survey (NHANES), a nationally representative sample of U.S. children and adolescents aged 3–19 years. By integrating accelerometer-derived PA metrics with BLL measurements, this study aims to [1] quantify the dose-response relationships between PA volume/intensity and BLLs, and [2] identify thresholds for lead exposure risk mitigation. Our findings could inform targeted interventions to maximize PA’s health benefits while minimizing lead-related harms in young populations.

## Materials and methods

### Study population

**Fig. 1:**
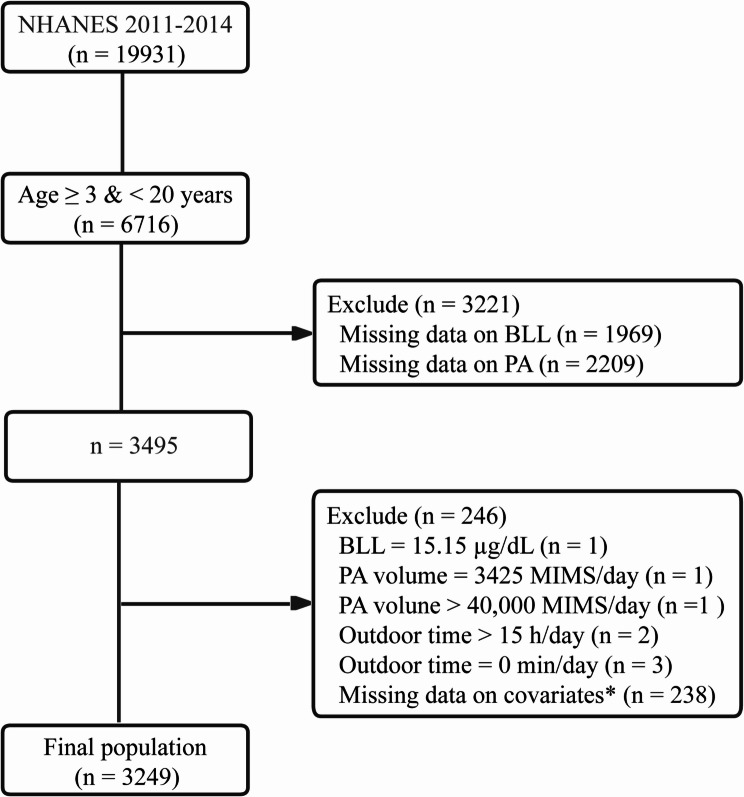
Flowchart of participant selection. *Covariates included age, sex, race/ethnicity, body mass index, family poverty-income ratio, and outdoor time. PA, physical activity (volume and intensity); BLL, blood lead level; NHANES, National Health and Nutrition Examination Survey; MIMS, monitor-independent movement summary units; Outdoor time, the amount of time spent outdoors

This cross-sectional analysis utilized data from two consecutive biennial cycles of the NHANES 2011–2012 and 2013–2014. NHANES data are publicly released in two-year cycles, and combining these periods allowed for a robust sample size while maintaining temporal consistency for the study. Participants aged 3–19 years were included, with exclusions applied hierarchically (Fig. 1). From the initial sample of 19,931 individuals, 6,716 met the age criteria. Participants were excluded for missing BLL measurements (*n* = 1,969) or PA data (*n* = 2,209), resulting in 3,495 eligible individuals. Further exclusions removed eight extreme outliers—one participant with a BLL of 15.15 µg/dL (far exceeding the 97.5th percentile of 1.48 µg/dL), two with implausible PA volumes (3,425 and 44,033 MIMS/day), and five with implausible outdoor time reports (0 min/day or > 15 h/day)—along with 238 participants missing key covariate data (age, sex, race/ethnicity, body mass index (BMI), family poverty-income ratio (PIR), and outdoor time). The final analytical cohort included 3,249 participants, with 74 (age ≥ 12 years) reporting active smoking episodes in the preceding 5-day period. Ethical approval was obtained from the National Center for Health Statistics Research Ethics Review Board (Protocol #2011-17).

### Variables

#### PA (exposure)

For the PA monitoring component, ActiGraph model GT3X + accelerometers (Pensacola, FL, USA) were utilized to measure both body movement acceleration and ambient light levels. Participants wore an accelerometer wristband on their nondominant hand during the examination visit. They were instructed to wear the wristband continuously for 24 hours a day, 7 days a week, spanning from the second to the eighth calendar days, and to remove it on the morning of the 9th day. Data from the initial and final partial days of wear were excluded prior to calculating the cumulative monitor-independent movement summary units (MIMS) metrics [19]. Acceleration measurements were recorded on the x-, y-, or z-axes (triaxial) and expressed in MIMS per minute. Only ‘wear’ minutes, encompassing both ‘wake’ and ‘sleep,’ were included in the analysis. Participants who wore the device for more than 10 h per day (both “wake” and “sleep”) and had at least 3 h of “sleep” and 7 h of “wake” wear, respectively, for a minimum of 3 valid days, were included in the analysis [19, 20].

The MIMS metrics utilized in this study encompassed PA volume, expressed as average MIMS per day, and PA intensity, represented by peak 60-minute MIMS. PA volume, denoted as average MIMS per day, quantified the total daily movement volume, calculated as the average daily MIMS per minute. The MIMS volume for each day was calculated by summing all MIMS per minute data accumulated within that day, and then averaging these values across all valid assessment days [21, 22]. The peak 60-minute MIMS value represents the mean of the top 60 MIMS per minute (not necessarily consecutive) across all valid days, indicating the intensity of movement during peak periods. This value was determined by ranking an individual’s MIMS per minute for each valid observation day, computing the mean of the top 60 values within each day, and then averaging the resulting MIMS per minute throughout all days of valid wear [19, 23]. The use of peak 60-minute MIMS corresponds to the recommended daily aerobic moderate-to-vigorous PA levels for children and adolescents [24] and partially corresponds to the peak 60-minute stepping cadence utilized in previous studies [19].

#### BLL (outcome)

Before distributing accelerometers for PA testing, whole-blood specimens were collected by certified phlebotomists through venipuncture into EDTA-coated, trace-metal-free polypropylene tubes at NHANES Mobile Examination Centers. Immediately after collection, samples were frozen at − 30 °C and shipped on dry ice to the CDC’s National Center for Environmental Health for analysis. Upon receipt, each specimen was diluted in a 1:1:48 volume ratio of whole blood: ultrapure water: diluent, where the diluent comprised 1% tetramethylammonium hydroxide, 0.5% EDTA, 10% ethanol, and 0.05% Triton X-100. Lead concentrations were quantified by inductively coupled plasma–dynamic reaction cell mass spectrometry (ICP-DRC-MS). The limit of detection (LOD) for BLL was 0.07 µg/dL. Measurements below the LOD were imputed using the LOD divided by the square root of 2. Elevated BLLs were identified using the CDC’s 2021 reference value of 3.5 µg/dL [25].

### Covariates

The demographic variables included in our analysis were age, sex, race/ethnicity (Mexican American, other Hispanic, non-Hispanic white, non-Hispanic black, and others), and the family poverty–income ratio (PIR). Body mass index (BMI) was calculated by dividing weight (kg) by height (m) squared. Furthermore, the amount of time spent outdoors (i.e. outdoor time) was considered a potential factor influencing BLL. Outdoor time was assessed using ambient light levels recorded by ActiGraph model GT3X + accelerometers (Pensacola, FL, USA). To estimate outdoor time, differentiation was made between time spent outdoors and indoors based on ambient light thresholds: ≥240 lx indicated outdoor settings, while < 240 lx indicated indoor settings [20, 26]. This threshold of 240 lx demonstrated 97% accuracy in distinguishing between indoor and outdoor conditions in a real-world setting [26]. The daily outdoor time per individual (minutes/day) was computed by averaging the daily outdoor minutes across valid wear days.

### Statistical analysis

All analyses employed a complex weighted-sampling design in accordance with NHANES guidelines (https://wwwn.cdc.gov/nchs/nhanes/tutorials/default.aspx). Summary statistics were computed for outcomes, exposures, and covariates. Chi-square and Wilcoxon rank-sum tests assessed baseline participant characteristics, stratified by sex. BLLs were ln-transformed (ln-BLLs) due to skewed distribution. Outdoor time was categorized into four quartiles (Q1, Q2, Q3, and Q4) for analysis as categorical variables. Weight status was ascertained using the 2000 Centers for Disease Control and Prevention (CDC) growth charts for children and adolescents [27]. Obesity was defined as having a BMI at or above the 95th percentile of the sex-specific BMI-for-age, while overweight was classified as a BMI falling between the 85th and 95th percentiles. Normal weight was designated for those with a BMI between the 5th and 85th percentiles, and underweight was characterized by a BMI less than the 5th percentile. Model 1 adjustments included age, sex, race/ethnicity, BMI category, and family PIR. Model 2 was adjusted for all variables in Model 1 plus outdoor time.

Firstly, Weighted multivariable linear regression analyses examined the associations of PA volume and intensity with BLL. Weighted multivariate generalized additive models (GAMs) with smoothing terms were further employed to assess any potential nonlinear relationships. In cases where a nonlinear relationship was detected, a weighted two-piecewise linear regression model was utilized to calculate the threshold effect of PAs on BLL, as illustrated in the smoothing plot. Additionally, to explore potential heterogeneities between sexes, we performed stratified weighted multivariable linear regression analyses and GAMs for males and females separately. Two adjusted models were applied in the weighted multivariable linear regression analyses and GAMs.

Secondly, the independent effect of outdoor time on the outcome was examined while controlling for PA. Specifically, outdoor time was analyzed as both a continuous variable and categorized into quartiles in weighted multivariable linear regression models, with adjustment for covariates in Model 1 (including PA). Subsequently, mediation analyses were conducted to estimate the potential mediating effects of outdoor time on the associations between PA and BLL. These mediation models also included covariates from Model 1. Direct and indirect effect pathways, along with the proportion of mediation, were quantified using nonparametric bootstrapping (*n* = 1,000) to assess statistical significance and effect magnitude.

Thirdly, Bayesian kernel machine regression (BKMR) was used to assess the joint effect of PAs and outdoor time on BLL. BKMR is proposed as a novel strategy for evaluating complex environmental contaminants [28]. The BKMR model specifically investigated the impact of exposure levels at quartiles relative to the medians. Model 1 was chosen for adjustment in the BKMR analysis.

Finally, we conducted two sensitivity analyses to test the robustness of our findings. First, recognizing that only 32 participants (1.0%) had elevated BLLs (> 3.5 µg/dL) and that the relationship between PA and BLL might differ at high exposure levels, we excluded all individuals with BLLs above 3.5 µg/dL and re-estimated the associations between PA volume, PA intensity, and ln-BLL. Second, because active smoking (*n* = 74, age ≥ 12 years) is a known determinant of BLLs, we removed all self-reported smokers and repeated the same multivariable regression and GAM analyses. Both sensitivity analyses employed the same survey weights and covariate adjustments as Model 2.

All significance levels were set to 0.05 in this study. Analyses were performed using R version 4.2.0 (http://www.R-project.org). BKMR, GAMs and mediation analysis were implemented with the R packages “bkmr” (version 0.2.2), “mgcv” (version 1.9–0), and “mediation” (version 4.5.0), respectively.

## Results

### Basic characteristics of study population

In the final analytical cohort (*n* = 3,249; 51% male; mean age 11.2 years), BLLs had a median of 0.6 µg/dL (range 0.12–8.75), PA volume averaged 17,118 MIMS/day (range 5,368–32,468), peak 60-minute PA intensity averaged 56.3 MIMS/min (range 24.1–109.2), and outdoor time averaged 107.8 min/day (range 0.1–448.1). The central tendency and variability of these four variables (means ± SD or medians [IQR]) are summarized in Table 1, whereas the full observed ranges were calculated separately for this report. Age-stratified distributions for BLL, PA volume, PA intensity, and outdoor time are depicted in Supplementary Fig. S1.

Sex-stratified comparisons (Table 1) revealed males had marginally higher BLLs (median: 0.6 vs. 0.5 µg/dL; *p* < 0.05), greater PA intensity (mean: 58.7 vs. 53.9 MIMS/min; *p* < 0.05), and longer outdoor time (mean: 112.6 vs. 102.9 min/day; *p* < 0.05). No significant sex differences were observed in PA volume, age, race/ethnicity, PIR, BMI category, or outdoor time category (all *p* > 0.05).

**Table 1:** Study participants and baseline characteristics

Characteristic	Overall (*n* = 3249, 100%)^*^	Male (*n* = 1650, 51%)^*^	Female (*n* = 1599, 49%)^*^	*P* value^#^
**Age (years)**	11.2 ± 4.3	11.2 ± 4.3	11.3 ± 4.3	0.4
**Race/ethnicity**				> 0.9
*Mexican American*	709 (16%)	338 (16%)	371 (17%)	
*Other Hispanic*	337 (7.4%)	174 (7.6%)	163 (7.3%)	
*Non-Hispanic White*	822 (54%)	439 (54%)	383 (53%)	
*Non-Hispanic Black*	873 (14%)	454 (14%)	419 (14%)	
*Other/multiracial*	508 (8.7%)	245 (8.6%)	263 (8.9%)	
**BMI category**				0.5
*Under weight*	91 (2.9%)	52 (3.5%)	39 (2.3%)	
*Normal weight*	1,890 (60%)	971 (60%)	919 (60%)	
*Overweight*	546 (17%)	261 (17%)	285 (16%)	
*Obese*	722 (21%)	366 (20%)	356 (22%)	
**Family PIR**	2.35 ± 1.63	2.41 ± 1.64	2.29 ± 1.62	0.2
**BLL (µg/dL)**	0.6 (0.4, 0.8)	0.6 (0.5, 0.9)	0.5 (0.4, 0.7)	**< 0.001**
**PA volume** **(MIMS/day)**	17,118 ± 4,078	17,198 ± 4,277	17,036 ± 3,862	0.5
**PA intensity** **(MIMS/min)**	56.3 ± 13.4	58.7 ± 14.5	53.9 ± 11.6	**< 0.001**
**Outdoor time (min/day)**	107.8 ± 69.3	112.6 ± 72.3	102.9 ± 65.8	**0.010**
**Outdoor time (quartiles)**				0.2
*1st ≤ 56.6*	941 (25%)	430 (23%)	511 (27%)	
*2nd 56.7–95.4*	884 (25%)	440 (25%)	444 (25%)	
*3rd 95.5 -144.7*	770 (25%)	403 (26%)	367 (24%)	
*4th ≥ 144.8*	654 (25%)	377 (26%)	277 (23%)	

^*^Mean ± SD; n (unweighted) (%); Median (IQR)

^#^Wilcoxon rank-sum test for complex survey samples; chi-squared test with Rao & Scott’s second-order correction

PA, physical activity; BMI, body mass index; BLL, blood lead level; PIR, poverty-income ratio; MIMS, monitor-independent movement summary units; Outdoor time, the amount of time spent outdoors; IQR, interquartile range

### Associations between PAs and BLL

**Fig. 2:**
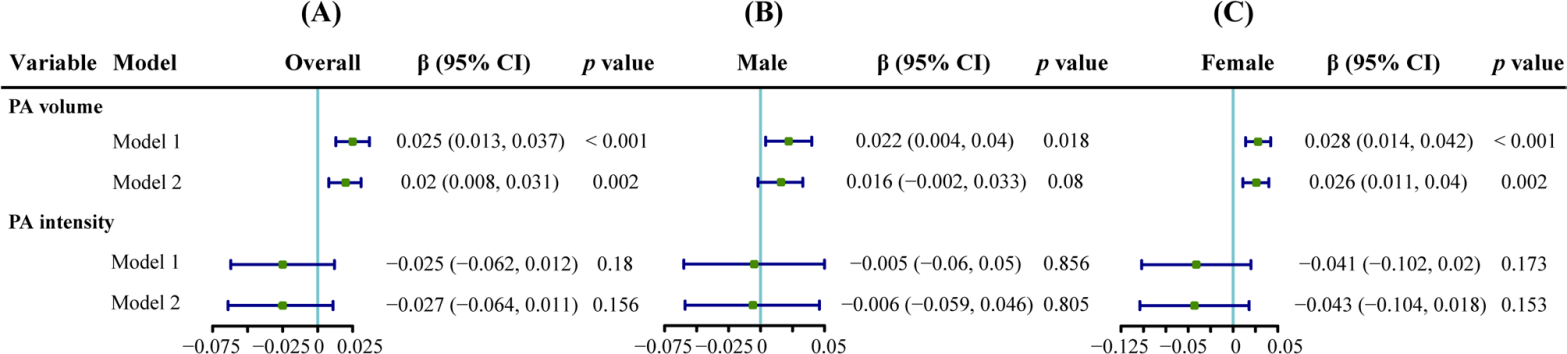
Multivariate regression results by group. The forest plot displays the results of the multivariate linear regression analysis for the (**A**) overall, (**B**) male, and (**C**) female groups. The model estimates depict the unit changes in ln-transformed blood lead level for each 1,000 MIMS/day increase in PA volume and for each 10 MIMS/min increase in PA intensity. Model 1 was adjusted for age, sex, race/ethnicity, classification of body mass index, and family poverty–income ratio. Model 2 was further adjusted for the amount of time spent outdoors based on Model 1. PA, physical activity; CI, confidence interval; MIMS, monitor-independent movement summary units

**Fig. 3:**
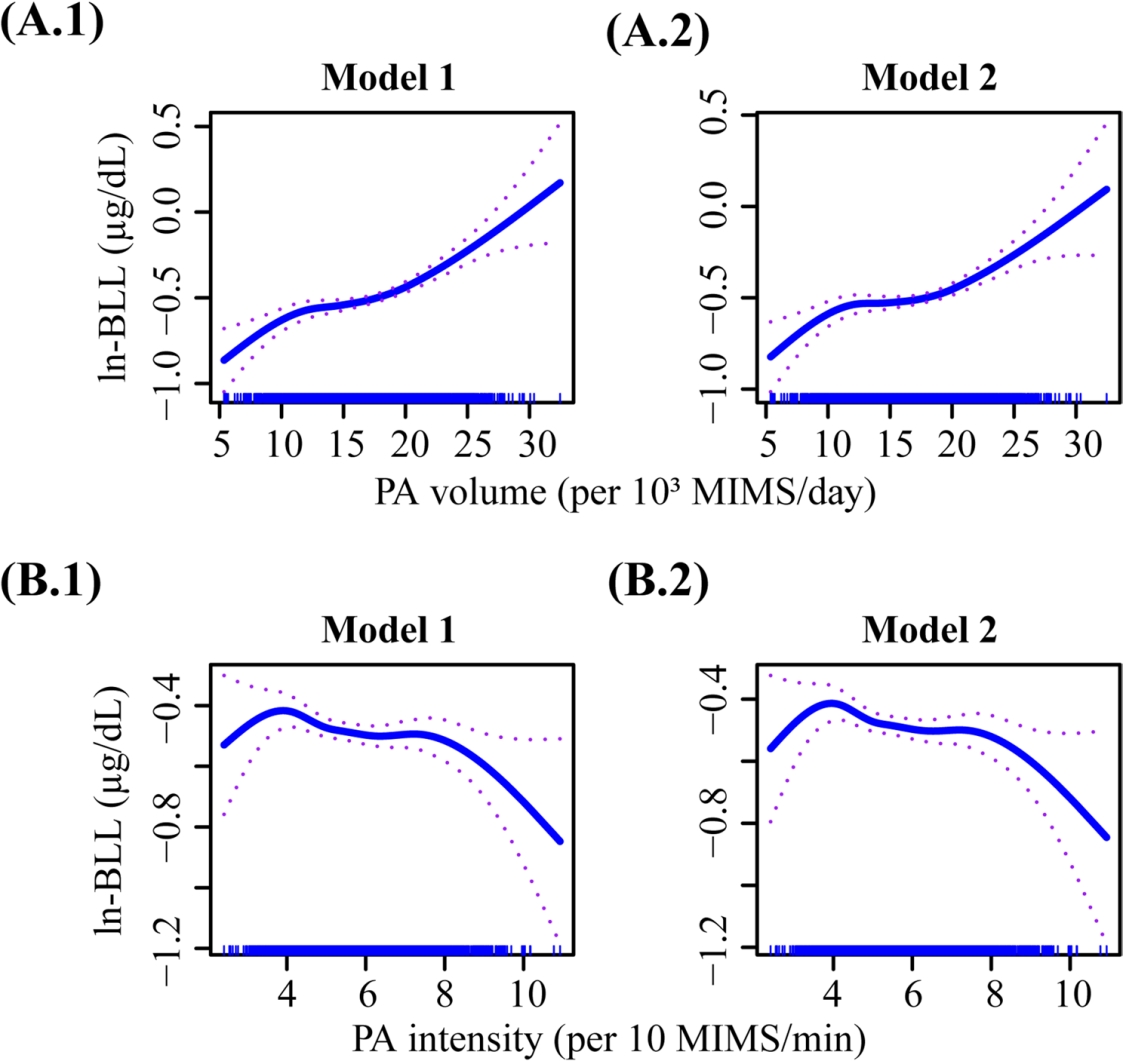
Nonlinear Relationships of PA volume and intensity with ln-BLL. The nonlinear relationship graph depicts (**A.1** and **A.2**) PA volume and (**B.1** and **B.2**) PA intensity with ln-BLL. Model 1 was adjusted for age, sex, race/ethnicity, classification of body mass index, and family poverty–income ratio. Model 2 was further adjusted for the amount of time spent outdoors based on Model 1. PA, physical activity; ln-BLL, ln-transformed blood lead level; MIMS, monitor-independent movement summary units

As depicted in Fig. 2A, weighted multivariable linear regression analyses revealed a significant positive correlation between PA volume and ln-BLL, regardless of whether model 1 (β = 0.025; 95% CI = 0.013 to 0.037; *p* < 0.001) or model 2 (β = 0.02; 95% CI = 0.008 to 0.031; *p* = 0.002) was used for adjustment. Conversely, PA intensity showed no statistical correlation with ln-BLL under both adjusted models. After accounting for outdoor time in Model 2, PA volume and PA intensity reduced the increase in ln-BLL compared with Model 1 without the adjustment for outdoor time. As shown in Fig. 3, the nonlinear relationship between PAs and ln-BLL fitted by GAMs indicated a significant nonlinear positive correlation for PA volume with ln-BLL (*p* < 0.001 for both models). Meanwhile, although PA intensity decreased the ln-BLL, it was not statistically significant (model 1: β = -0.025, 95% CI = -0.062 to 0.012, *p* = 0.18; model 2: β = -0.027, 95% CI = -0.064 to 0.011, *p* = 0.156). A weighted two-piecewise linear regression model was used to calculate the threshold effect of PA volume on BLL based on the smoothing plot. As presented in Table 2, results showed that after adjusting for the impact of outdoor time, PA volume did not significantly increase BLL below the threshold point of 19,700 MIMS/day. However, above the threshold point of 19,700 MIMS/day, every increase of 1000 MIMS/day resulted in an increase of 0.041 (95% CI = 0.019 to 0.063; *p* = 0.001) in the ln-BLL.

**Table 2:** Results of weighted two-piecewise linear regression model between PA volume and ln-BLL

Outcomes	Model1^*^	Model 2^#^
	β (95% CI), *P*	β (95% CI), *P*
Line model	0.025 (0.013, 0.037), < 0.001	0.02 (0.008, 0.031), 0.002
Threshold point (10^3^ MIMS/day)	19.7	19.7
< Threshold point	0.018 (0.004, 0.031), 0.013	0.012 (-0.003, 0.027), 0.106
> Threshold point	0.046 (0.021, 0.071), 0.001	0.041 (0.019, 0.063), 0.001
*P* for likelihood ratio test^£^	0.048	0.044

^*^ Model 1 was adjusted for age, sex, race/ethnicity, BMI category, and family poverty-income ratio.

^#^ Model 2 was further adjusted for the amount of time spent outdoors based on Model 1.

^£^*P* value for likelihood ratio test which indicated that the relationships between PA volume and ln-transformed blood lead level exhibited a threshold effect.

PA, physical activity; CI, confidence interval; MIMS, monitor-independent movement summary units

### Associations between outdoor time and BLL

As shown in Table 3, both continuous and categorical analyses revealed significant associations between outdoor time and BLLs, adjusted for PA volume and intensity. When outdoor time was modeled as a continuous variable, each additional hour per day of outdoor time was associated with a 0.03 µg/dL increase in ln-BLL (95% CI: 0.02–0.05; *p* < 0.001) in both PA volume-adjusted and PA intensity-adjusted models.

For categorical analyses, participants in the highest quartile of outdoor time (Q4) exhibited the strongest association, with a 0.13 µg/dL increase in ln-BLL compared to the reference group (Q1) in the PA volume-adjusted model (95% CI: 0.08–0.19; *p* < 0.001). Similar results were observed in the PA intensity-adjusted model (Q4: β = 0.13, 95% CI: 0.07–0.18; *p* < 0.001). A significant dose-response relationship was evident, with monotonically increasing β values across quartiles (Q3: β = 0.06, *p* = 0.024 in PA volume-adjusted model; Q3: β = 0.05, *p* = 0.046 in PA intensity-adjusted model). Both models demonstrated robust linear trends (P for trend < 0.001), reinforcing the graded association between prolonged outdoor exposure and elevated BLLs.

**Table 3:** Associations between outdoor time with ln-BLL

Outdoor time	Volume-adjusted model^*^			Intensity-adjusted model^#^	
	β (95% CI)	*P*		β (95% CI)	*P*
Contineous (per h/day)	0.03 (0.02,0.05)	< 0.001		0.03 (0.02,0.05)	< 0.001
Quartiles (min/day)					
Q1 (≤ 56.6)	Ref.	—		Ref.	—
Q2 (56.7–95.4)	0.01 (-0.04, 0.06)	0.7		0.00 (-0.05, 0.05)	0.9
Q3 (95.5 -144.7)	0.06 (0.01, 0.11)	0.024		0.05 (0.00, 0.11)	0.046
Q4 (≥ 144.8)	0.13 (0.08, 0.19)	< 0.001		0.13 (0.07, 0.18)	< 0.001
*P*trend^£^	< 0.001			< 0.001	

^*^ Model was adjusted for age, sex, race/ethnicity, BMI category, family poverty-income ratio, and PA volume.

^#^ Model was adjusted for age, sex, race/ethnicity, BMI category, family poverty-income ratio, and PA intensity.

^£^ Ptrend tests for linear trend across outdoor time quartiles.

PA, physical activity

### Mediating effect of outdoor time on the relationship between PAs and BLL

In the mediation analysis, outdoor time significantly mediated the relationship between PA volume and ln-BLL (mediation effect, 0.008; 95% CI = 0.004 to 0.012; direct effect, 0.017; 95% CI = 0.006 to 0.028), whereas no such mediation was observed for the relationship between PA intensity and ln-BLL (Table 4). The proportion of the mediation effect of outdoor time as a mediator pathway for PA volume with ln-BLL was 32.8%.

**Table 4:** Proportions of PA associations with ln-BLL mediated by the amount of time spent outdoors^*^

	PA volume (per 10^3^ MIMS/day)	PA intensity (per 10 MIMS/min)
Total effect (95% CI)	0.025 (0.014, 0.036)	-0.025 (-0.061, 0.009)
Direct effect (95% CI)	0.017 (0.006, 0.028)	-0.022 (-0.058, 0.011)
Mediated effect (95% CI)	0.008 (0.004, 0.012)	-0.003 (-0.007, 0.002)
Proportion on mediated (%)^#^	32.8	.

^*^ Model was adjusted for age, sex, race/ethnicity, classification of body mass index, and family poverty-income ratio.

^#^ Proportion mediated was not estimated when the mediated effect was nonsignificant.

PA, physical activity; CI, confidence interval; MIMS, monitor-independent movement summary units

### BKMR analyses

**Fig. 4:**
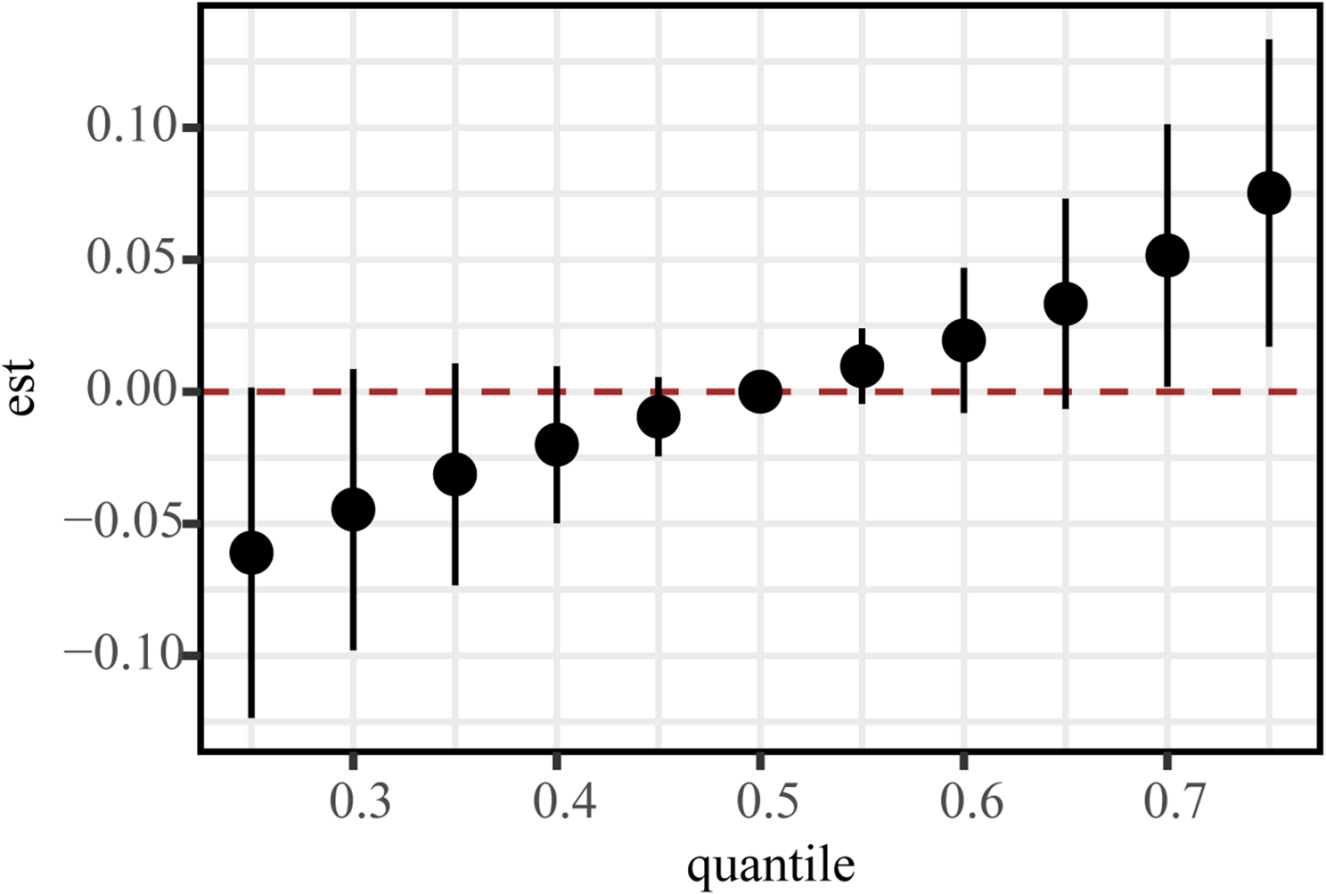
Joint effects of PA volume, intensity, and outdoor time on ln-BLL. The figure illustrates the estimated change in ln-transformed blood lead level when the three exposures were set at specific percentiles (ranging from the 25th to 75th percentiles) compared with the situation when all exposures are at their 50th percentile. Model was adjusted for age, race/ethnicity, classification of body mass index, and family poverty–income ratio. PA, physical activity

In the BKMR model, a cumulative effect of increasing trend was found for co-exposures of PAs and outdoor time. The overall effect was statistically significant when the co-exposures were at or above their 70th percentile compared with the situation when all co-exposures were at their median values (Fig. 4).

### Stratified analyses by sex

**Fig. 5:**
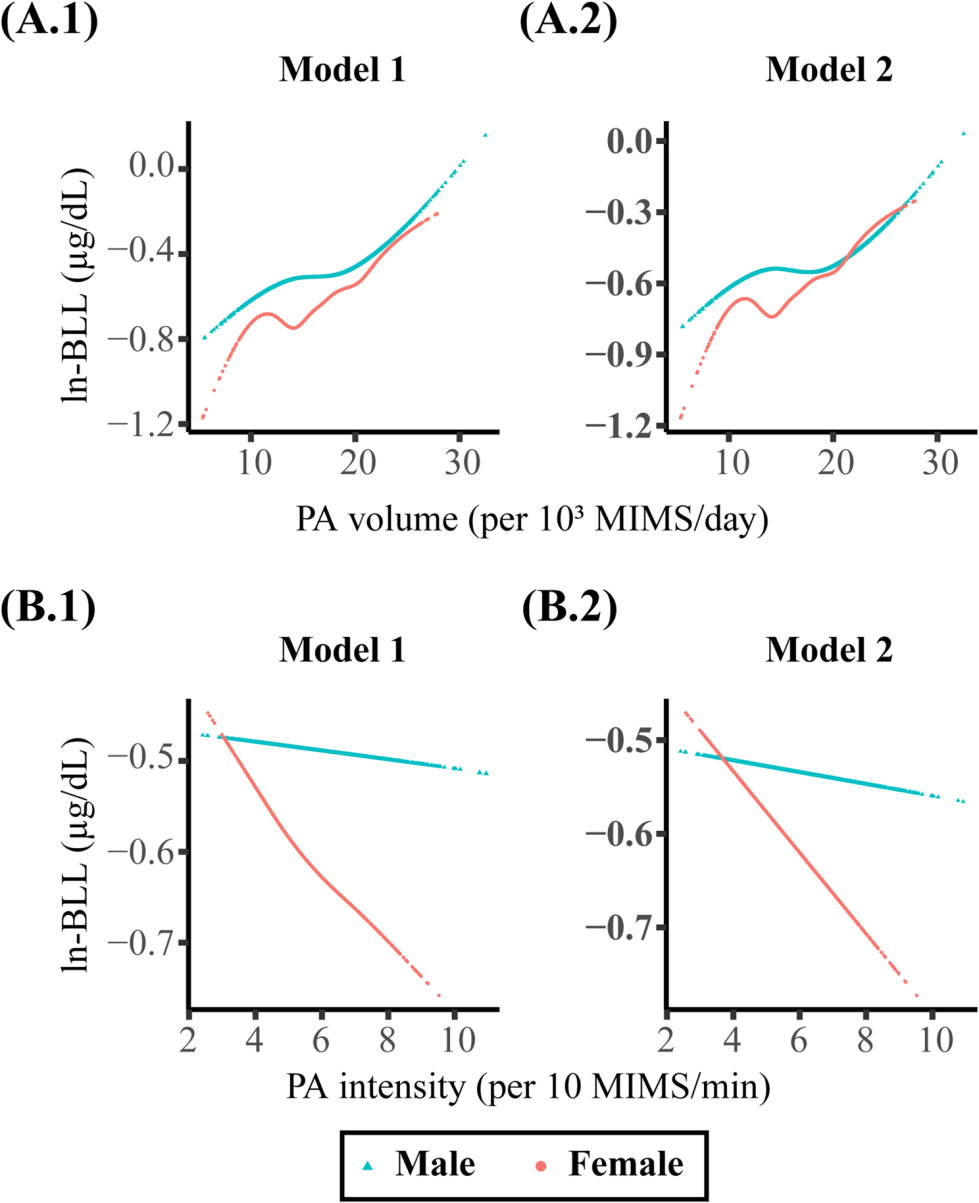
Sex-stratified nonlinear associations of PA volume and intensity with ln-BLL. The nonlinear relationships between PA volume (**A.1** and **A.2**) and PA intensity (**B.1** and **B.2**) with ln-BLL are illustrated. Model 1 was adjusted for age, race/ethnicity, classification of body mass index, and family poverty–income ratio. Model 2 was further adjusted for the amount of time spent outdoors based on Model 1. PA, physical activity; ln-BLL, ln-transformed blood lead level; MIMS, monitor-independent movement summary units

Results of subgroup analysis based on sex were consistent with the trends observed in the overall analysis. A significant interaction existed between sexes. As shown in Figs. 2B-C and 5A.1–2, at the same level of PA volume, the ln-BLL in the female group was significantly lower than that in the male group in both adjusted models (all interaction *p* < 0.001). When PA intensity was at the same level, the reduction in ln-BLL in the female group was significantly greater than in the male group, regardless of the adjusted models (all interaction *p* < 0.01). Results are shown in Figs. 2B-C and 5B.1–2.

### Sensitivity analysis

A total of 3,217 participants had normal BLLs (≤ 3.5 µg/dL) and only 32 participants had high BLLs (> 3.5 µg/dL). Considering the potential impact of the small number of elevated BLL data on the results, a sensitivity analysis was performed to assess the stability of the association between PAs and BLL by excluding participants with high BLLs. The linear and nonlinear correlations between PAs and BLL after excluding participants with high BLLs were consistent with those before exclusion. Sensitivity analysis revealed a threshold effect of PA volume on ln-BLL, with a threshold point at 19,600 MIMS/day. This finding was essentially consistent with the threshold point of 19,700 MIMS/day before excluding participants with high BLLs (Supplementary Table S1 and Fig. S2).

After excluding the 74 active smokers, the analysis was repeated in the remaining 3,159 participants. The positive association between PA volume and ln-BLL was marginally attenuated but remained statistically significant: β = 0.023 (95% CI: 0.010–0.036, *p* = 0.001) in the unadjusted model and β = 0.018 (95% CI: 0.005–0.030, *p* = 0.007) after adjusting for outdoor time. In contrast, PA intensity retained a negative association with ln-BLL (β < 0), but this relationship did not reach statistical significance (*p* > 0.05; Supplementary Fig. S3). These sensitivity results closely mirrored those from the full cohort (including active smokers), suggesting that smoking status did not substantially confound the observed associations.

## Discussion

This study was designed to elucidate the association between BLL and PAs (volume and intensity) among a large cohort of participants. Our findings revealed a significant positive correlation between PA volume and ln-BLL, whereas the relationship between PA intensity and ln-BLL, although negatively correlated, did not reach statistical significance. Their combined effect ultimately results in an overall increase in BLL. We also observed significant sex differences in the association between PA and ln-BLL.

The sources of lead pollution in the air include automobile exhaust, non-ferrous metal production, waste incineration, coal combustion, soil, dust, and building coatings [29]. Over time, developed countries have significantly reduced the use of lead. This finding is consistent with our data, indicating that only about 1% of participants have BLLs exceeding the reference value (3.5 µg/dL). Despite observed declines in children’s BLLs in the United States over the past few decades, lead exposure reservoirs still persist, primarily in soil, where remnants of leaded gasoline and dust from various sources continue to accumulate [29, 30]. In summary, evidence suggests that lead exposure persists as a public health issue across both high-income and low- to middle-income countries [31]. Once lead enters the atmosphere, it can be inhaled, potentially increasing BLLs. Studies by Richmond–Bryant et al. [32] and Zahran et al. [7] confirmed this process, each demonstrating the impact of atmospheric lead concentrations on BLLs in the general population and children, respectively.

Our study observed a positive association between daily PA volume and BLL, with a notable threshold effect. When outdoor time was unadjusted, an approximately 2.6-fold increase in ln-BLL was observed when PA volume reached the threshold point (19,700 MIMS/day). After adjusting for outdoor time in the model, the below-threshold association was attenuated to nonsignificance (*p* > 0.05), whereas the above-threshold effect remained robust (β = 0.041, 95% CI 0.019–0.063, *p* = 0.001). This divergence suggests two distinct exposure pathways. At PA volumes below ~ 19,700 MIMS/day, increases in BLL are largely mediated by outdoor lead inhalation—our mediation analysis showed that outdoor time explains 32.8% of the PA volume–BLL relationship (Table 3)—so adjusting for outdoor time removes much of the apparent volume effect. By contrast, at higher PA volumes, additional factors come into play: elevated ventilation rates boost overall pollutant uptake (including indoor-resuspended dust) [7]. These mechanisms operate independently of measured outdoor time, preserving the positive association above the threshold. Previous studies have reported similar results. A study in Korea found that the odds of having BLLs exceeding 2.76 µg/dL were greater in the high PA group compared with the low PA group [13]. Another study in Italy found slightly higher BLLs in urban runners than in rural runners and non-runners [33]. These studies, however, did not explore the threshold effect observed here.

Both continuous and categorical analyses of outdoor time demonstrated significant associations with BLLs, after adjusting for PA volume and intensity. To elucidate the role of outdoor time in the relationship between PA volume and BLL, we performed a mediation analysis. We found that 32.8% of the increase in ln-BLL could be attributed to the mediating effect of outdoor time (*p* < 0.05). Furthermore, a cumulative effect of increasing trend was found for co-exposures of PAs and outdoor time in the BKMR model. The overall effect was statistically significant when the co-exposures were at or above their 70th percentile compared with the situation when all co-exposures were at their median values. Our comprehensive analysis suggests the pathway of lead inhalation through outdoor activities leading to elevated BLLs. Nevertheless, the positive correlation between PA volume and ln-BLL (β = 0.02; 95% CI = 0.008–0.031) remained significant even after adjusting for outdoor time. This indicates that indoor exercise environments, particularly those with lead-containing paint and dust [30, 34, 35], should not be overlooked.

Our results indicated an inverse correlation between PA intensity (peak 60-minute MIMS) and ln-BLL, although not statistically significant (*p* > 0.05). Compared with the model without adjusting for outdoor time (β = -0.025; 95% CI = -0.062 to 0.012; *p* = 0.18), the adjusted model (β = -0.027; 95% CI = -0.064 to 0.011; *p* = 0.156) showed a greater decreasing trend. Previous studies have suggested that PA may reduce BLL through mechanisms such as sweating. Tang et al. [15] demonstrated that dynamic exercise can effectively remove toxic heavy metals, including lead, from the body, whereas Haber et al. [11] found that endurance training improved BLLs in lead-exposed workers, possibly through sweat or bile excretion. Kuan et al. [14] found that sweating induced by exercise is an effective way to reduce heavy metals (such as lead), further supporting this notion. These findings provide a reasonable explanation for our research outcomes. PA volume reflects cumulative daily activity, while the peak 60-minute MIMS metric serves as a proxy for exercise intensity. Higher-intensity exercise typically induces profuse sweating, which may enhance lead excretion through perspiration, whereas increased PA volume does not necessarily correlate with enhanced detoxification. Notably, prior studies have often combined PA volume and intensity into a single metric; our study is the first to independently examine their distinct relationships with BLLs from both volume and intensity perspectives, revealing differential exposure-detoxification dynamics.

Stratified sex analysis revealed significant interactions, with males showing higher BLLs at comparable PA volumes, while females exhibited a more pronounced decrease in BLLs at equivalent levels of PA intensity. One plausible explanation lies in gendered patterns of activity: in our cohort, girls spent significantly less time outdoors than boys (mean 102.9 vs. 112.6 min/day; *p* < 0.05), reflecting a tendency for girls spent significantly less time outdoors than boys—reflecting a tendency for girls to favor structured, predominantly indoor activities—while boys engaged in more unstructured outdoor play [36]. Because indoor settings generally exhibit lower resuspension and infiltration of coarse, soil-derived particulates—resulting in reduced airborne soil-lead concentrations—high-intensity exercise indoors may involve less inhaled lead than equivalent outdoor activity [37].

Beyond exposure differences, physiological factors may also contribute. Kuan et al. observed higher levels of heavy metal excretion in female sweat, which may partly explain our finding, suggesting that females may excrete more lead through sweating [14]. The Swedish Twin Registry suggests that female BLLs largely reflect genetic factors (37%), whereas male BLLs primarily reflect environmental exposure for over 97% [38]. Although the specific genes remain to be fully characterized, polymorphisms in the vitamin D receptor (VDR), δ-aminolevulinic acid dehydratase (ALAD), and hemochromatosis (HFE) genes have all been implicated in differential lead absorption, retention, and excretion [39, 40]. Additional factors—such as hormonal influences and body-composition differences—may also affect lead mobilization and clearance, as well as activity preferences, although further study is needed. Taken together, these behavioral and biological distinctions likely underlie the sex-specific associations we observed. Nonetheless, targeted mechanistic studies will be essential to confirm these hypotheses and to guide sex-tailored interventions.

We also conducted sensitivity analysis excluding participants with higher BLLs. Results showed that the correlation between PA and BLL in participants with low BLLs was consistent with that of all participants, indicating that the impact of PA on individuals with high or low BLLs may be consistent. Although the majority of participants in our study had low BLLs (99%), recognizing that even low levels of BLLs pose risks to the nervous, immune, and cardiovascular systems is important [41, 42].

While the modest increase in BLLs associated with higher PA volume is a potential concern, the extensive health advantages of PA for children and adolescents clearly outweigh this risk. Regular PA enhances cardiovascular fitness, lowers blood pressure, improves lipid and glucose metabolism [8], and supports bone mineralization and muscle development [24]. It also promotes mental well-being by reducing anxiety and depression while boosting self-esteem [43]. Additionally, even brief PA interventions can improve cognitive functions such as attention and executive function [44]. To maximize these benefits while minimizing lead exposure—primarily via inhalation of resuspended dust and ingestion of contaminated soil or dust—PA guidelines should emphasize higher-intensity activities in low-pollution settings. Simultaneously, public health strategies must integrate soil remediation and air-quality monitoring to safeguard children’s overall health [7, 45].

Our study’s strengths include the objective measurement of PA using accelerometers. The accelerometer provided minute-by-minute MIMS values, indicating exercise intensity. Additionally, it monitored light intensity per minute to distinguish between indoor and outdoor environments. This allowed us to analyze the relationship between PA and BLL across three dimensions—volume, intensity, and outdoor time—yielding clearer and more definitive results. Furthermore, by conducting BKMR analysis of the total effects of these three factors, performing multivariate linear regression and nonlinear regression analyses of individual variables, and exploring the role of outdoor time in the relationship between exercise and BLL through mediation analysis, the results mutually corroborated one another and thus enhanced their persuasiveness. Finally, sensitivity analysis confirmed the robustness of our study’s findings.

This study has several limitations. First, the cross-sectional design precludes causal inference between PA and BLLs, as temporal relationships remain unestablished. Second, unmeasured confounders—including environmental factors such as ambient lead concentrations during PA, housing age (years of residence), and residential location (urban vs. rural), as well as nutritional factors (e.g., serum calcium and iron levels)—may introduce residual bias. Third, wrist-worn ActiGraph GT3X + accelerometers, despite improving compliance, introduced measurement limitations, including inaccuracies in step-counting and intensity estimation for non-ambulatory movements (e.g., cycling), misclassification of upper-body activities, and variability due to device placement [46, 47]. Fourth, the MIMS metric lacks validated intensity thresholds or guideline-aligned cut-points, complicating comparisons with studies using traditional PA metrics (e.g., counts per minute). This gap limits direct comparisons with studies using traditional PA metrics and underscores the need to establish MIMS-based intensity cut-points in future research.

## Conclusion

In this nationally representative sample of U.S. youth, we demonstrate that cumulative daily PA volume and peak intensity exert opposing influences on blood lead levels. Above a threshold of approximately 19,700 MIMS/day, increased volume remains associated with higher BLL even after accounting for outdoor exposure, likely reflecting enhanced inhalation and nondifferentiated lead uptake. In contrast, higher PA intensity trends toward lower BLL, consistent with sweat-mediated lead elimination, and is most pronounced among females. These dual pathways suggest that public-health strategies should promote vigorous activity in low‐lead settings—thereby maximizing detoxification—while mitigating risks from excessive overall movement in contaminated environments. Future longitudinal and intervention studies are warranted to confirm causality, refine MIMS‐based intensity thresholds, and guide sex‐specific recommendations that optimize the benefits of children’s and adolescents’ PA without exacerbating lead exposure.

## Electronic supplementary material

Below is the link to the electronic supplementary material.

Supplementary Material 1

Supplementary Material 2

## Data Availability

Data is provided within the manuscript or supplementary information files.

## Abbreviations

PA: Physical activity
BLL: Blood lead level
NHANES: National Health and Nutrition Examination Survey
MIMS: Monitor-independent movement summary units
LOD: Limit of detection
BMI: Body mass index
PIR: Poverty-income ratio
GAM: Generalized additive model
BKMR: Bayesian kernel machine regression
CI: Confidence interval
