# Supplementary material for: Differential impacts of physical activity volume and intensity on blood lead levels in children and adolescents: a cross-sectional study
Source: BMC Public Health. 2025 Jun 2;25:2049. doi: 10.1186/s12889-025-23333-8 (PMC12131555; doi:10.1186/s12889-025-23333-8)
Supplement: Supplementary file 1 — Supplementary Material 1 [file 12889_2025_23333_MOESM1_ESM.docx]

**Table S1** **Results of weighted two-piecewise linear-regression model between PA volume and ln-BLL.**

| Outcomes | Model1**^*^** | Model 2^#^ |
| --- | --- | --- |
|  | β (95% CI), *P* value | β (95% CI), *P* value |
| Line model | 0.025 (0.016, 0.035), < 0.001 | 0.021 (0.011, 0.031), < 0.001 |
| Threshold point  (10³ MIMS/day) | 19.6 | 19.6 |
| < Threshold point | 0.019 (0.007, 0.032), 0.005 | 0.015 (0.002, 0.029), 0.031 |
| > Threshold point | 0.042 (0.027, 0.057), < 0.001 | 0.038 (0.023, 0.053), < 0.001 |
| *P* for likelihood ratio test^£^ | 0.047 | 0.049 |

**^*^**Model 1 was adjusted for age, sex, race/ethnicity, BMI category and family poverty-income ratio

^#^Model 2 was further adjusted for the amount of time spent outdoors based on Model 1.

^£^*P* value for likelihood ratio test which indicated that the relationships between PA volume and ln-transformed blood lead level exhibited a threshold effect.

PA, physical activity; ln-BLL, ln-transformed blood lead level; CI, confidence interval; MIMS, monitor-independent movement summary units; BMI, body mass index


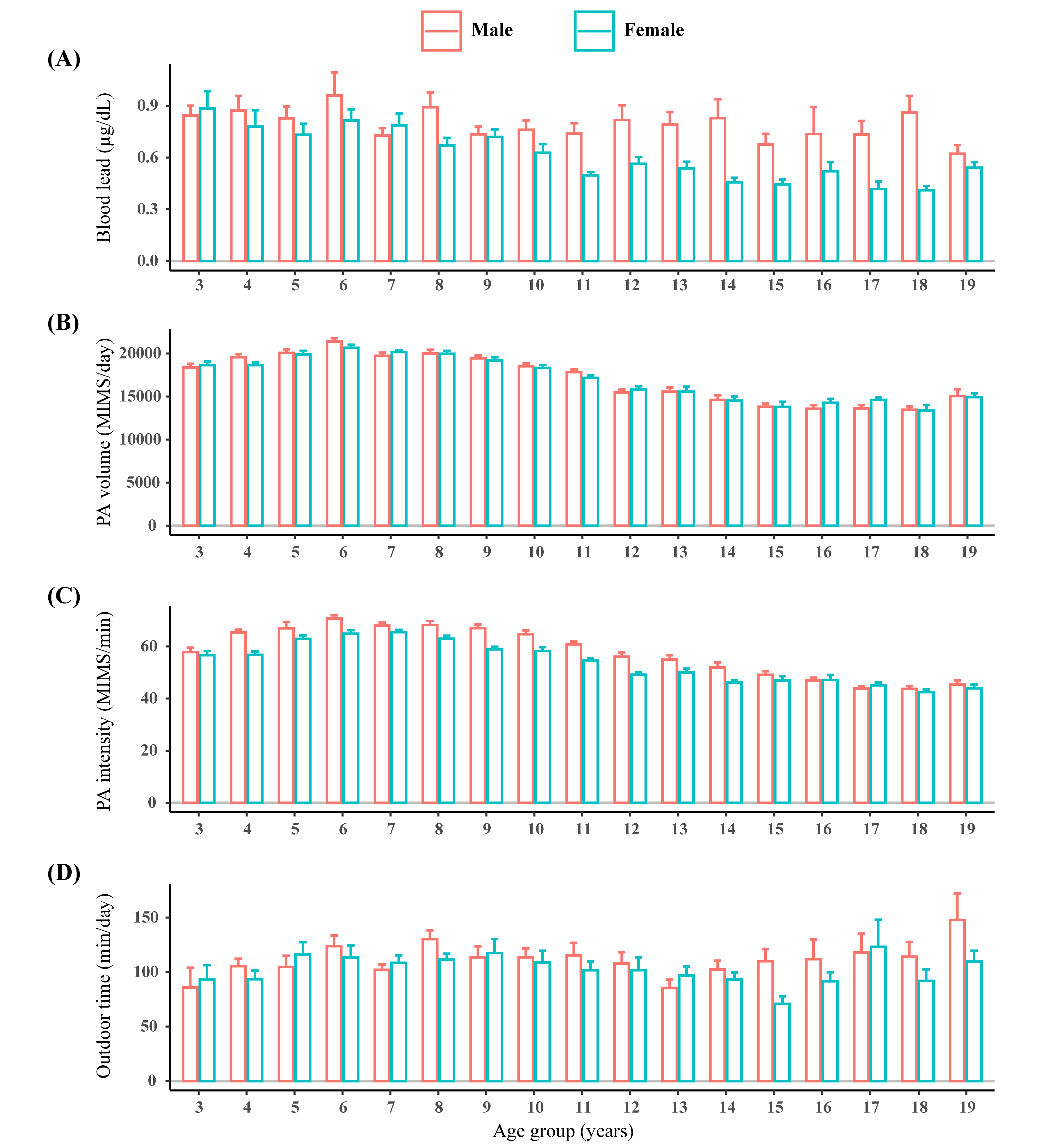


**Fig. S1 Distribution of variables across age groups**

Weighted means and standard errors are displayed for blood lead level (µg/dL), PA volume (MIMS/day), PA intensity (peak 60-minute MIMS), and outdoor time (min/day) in panels A, B, C, and D, respectively.

PA, physical activity; MIMS, monitor-independent movement summary units; Outdoor time, the amount of time spend outdoors


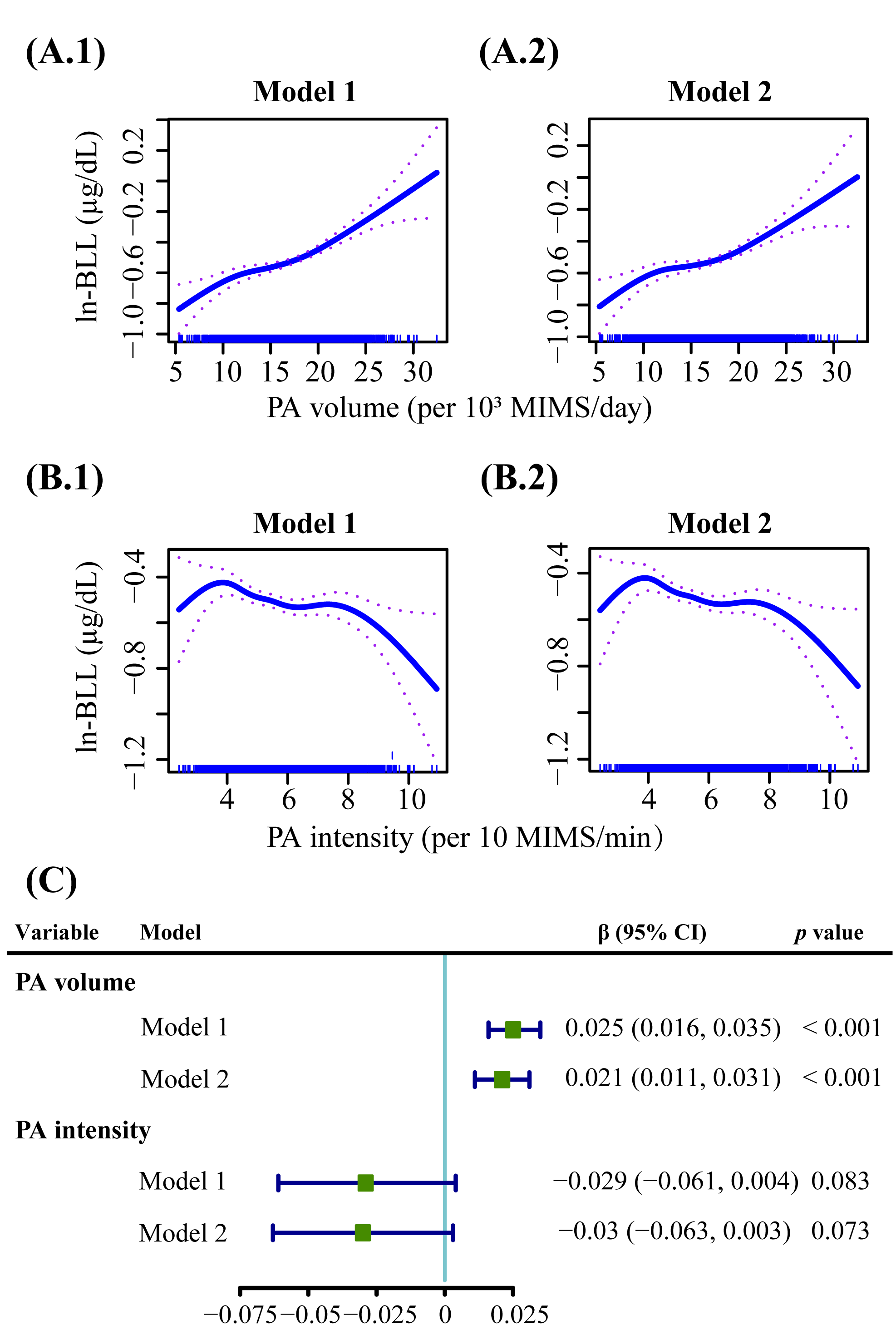


**Fig. S2 Sensitivity analysis results after excluding individuals with high BLLs.**

The nonlinear relationship graph illustrates (A.1 and A.2) PA volume and (B.1 and B.2) PA intensity with ln-ln-BLL. (C) Multivariate linear-regression results for PA volume (per 10^3^ MIMS/day) and PA intensity (per 10 MIMS/min) with ln-BLL, respectively. Model 1 was adjusted for age, sex, race/ethnicity, BMI category, and family poverty–income ratio. Model 2 was further adjusted for the amount of time spent outdoors based on Model 1.

PA, physical activity; ln-BLL, ln-transformed blood lead level; MIMS, monitor-independent movement summary units


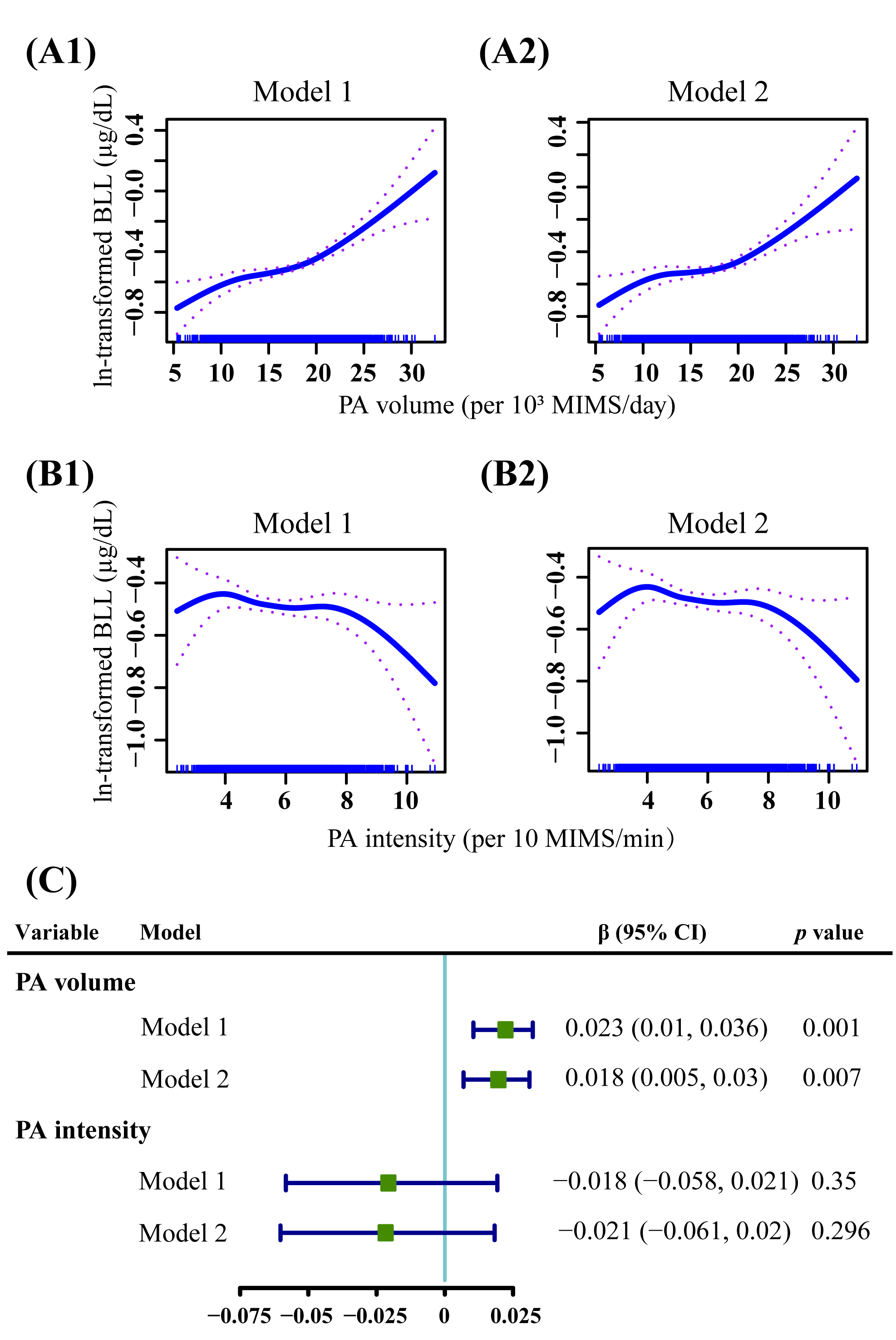


**Fig. S3 Sensitivity analysis results after excluding active smokers.**

The nonlinear relationship graph illustrates (A.1 and A.2) PA volume and (B.1 and B.2) PA intensity with ln-ln-BLL. (C) Multivariate linear-regression results for PA volume (per 10^3^ MIMS/day) and PA intensity (per 10 MIMS/min) with ln-BLL, respectively. Model 1 was adjusted for age, sex, race/ethnicity, BMI category, and family poverty–income ratio. Model 2 was further adjusted for the amount of time spent outdoors based on Model 1.

PA, physical activity; ln-BLL, ln-transformed blood lead level; MIMS, monitor-independent movement summary units
